# An Exploration of the Experiences and Perceptions of TBI Survivors About Accessing Vocational Rehabilitation During the COVID-19 Pandemic

**DOI:** 10.1155/2024/8414358

**Published:** 2024-07-30

**Authors:** Tarryn Petersen, Mogammad Shaheed Soeker

**Affiliations:** Occupational Therapy Department University of the Western Cape, Cape Town, South Africa

**Keywords:** barriers and facilitators to therapy, employment, return to work

## Abstract

**Background:** Statistics indicate a high prevalence of TBI in South Africa, with many individuals with TBI not returning to work. The lack of return to work among TBI survivors is particularly due to factors such as injury severity, preinjury educational and occupational status, and age at injury. However, in addition to the above factors, there was the COVID-19 pandemic, which resulted in the de-escalation of nonessential outpatient services in order to assist with curbing the spread of the virus.

**Objective:** The aim of the article is to explore the experiences and perceptions of TBI survivors about accessing vocational rehabilitation during the COVID-19 pandemic and how this has affected their worker roles.

**Method:** A descriptive, explorative qualitative research design was used, and semistructured interviews were conducted to collect data. The authors subsequently analysed the transcribed data using a thematic analysis approach. The COREQ (consolidated criteria for reporting qualitative research) checklist was used as a reporting guideline. Ten TBI survivors and two individuals working in the public health sector participated in this study. Two semistructured interviews were conducted with each research participant.

**Results:** Three themes emanated from the study, namely, Theme 1: “The barriers to accessing rehabilitation during the COVID-19 pandemic” represents the participants' barriers to accessing rehabilitation programmes throughout the COVID-19 pandemic. Theme 2: “Lack of rehabilitation negatively influenced the individual with TBI occupational performance” describes how the lack of OT rehabilitation during the COVID-19 pandemic impacted the participants' quality of life. Theme 3: “Factors that facilitated access to rehabilitation during the COVID-19 pandemic” describes the factors that facilitated access to OT rehabilitation services during the COVID-19 pandemic.

**Conclusion:** The study found that there were barriers and facilitators to accessing occupational therapy rehabilitation during the COVID-19 pandemic for TBI survivors. More research needs to be conducted to explore the efficacy of telehealth/telemedicine for occupational therapy rehabilitation and the role of the occupational therapist in global pandemics.

## 1. Introduction

According to Moller, Lingah, and Phehlukwayo [1], TBI frequently causes cognitive, behavioural, physical, and functional limitations that may affect the individual's independence in activities of daily living (ADLs), including work. Soeker [2] indicated that many TBI survivors are of working age and run the risk of becoming unproductive persons in the community who no longer consider work as an important facet of their lives [2]. The Society of Occupational Medicine [3] reported that the coronavirus (COVID-19) pandemic is acknowledged as a worldwide health emergency by the World Health Organization (WHO), and community health procedures have been employed, involving social distancing, work constraints, and promoting working from home. In response to the COVID-19 pandemic, nonessential rehabilitative services (including outpatient occupational therapy rehabilitation for TBI patients) were suspended to assist with curbing the spread of the virus. This resulted in TBI survivors being unable to complete their occupational therapy rehabilitation and, in turn, unable to return to their worker roles.

In South Africa, there is inadequate access to rehabilitation services within the public sector. The consequence for individuals with TBI is that very few obtain satisfactory rehabilitation services; only 16 of the 654 individuals with TBI in the 2009 audit of a tertiary hospital (Western Cape, South Africa) attended a public rehabilitation facility [4]. The current study therefore explored the experiences and perceptions of TBI survivors and their reintegration into their worker roles, with a specific focus on access to rehabilitation during the COVID-19 pandemic and how this influenced their ability to return to work. The research was conducted at the occupational therapy department of a tertiary hospital in the Western Cape. This study explored the importance of occupational therapy intervention in the facilitation of reintegration into work-related occupations for TBI survivors.

## 2. Literature Review

### 2.1. Epidemiology of TBI

According to the WHO, diagnostic criteria for TBI include confusion or disorientation, loss of consciousness for 30 min or less, posttraumatic forgetfulness for less than 24 h, and/or other short-term neurological irregularities such as focal signs, seizures, and intracranial lesions not requiring surgery, as well as a Glasgow Coma Scale score of 13–15 after 30 min postinjury or later upon presentation for healthcare services [5].

The National Institute for Occupational Health [6] stated that, in South Africa, researchers have also found that men account for a higher percentage of TBI victims than women, with a projected man-to-woman ratio of 4:1. Almost 50% of TBIs are caused by motor vehicle, bike, or pedestrian-car accidents. The second most common cause of TBI is attributed to falls, which are more frequent among younger people. Incidents related to violence account for roughly 20% of TBIs, which are almost equally distributed among firearm and nonfirearm attacks.

TBI frequently results in neurocognitive deficits (such as impaired awareness, failure to develop visuospatial associations, or inadequate executive function) and psychological health concerns; for example, 30%–70% of individuals with TBI are diagnosed with depression. TBI survivors also demonstrate increased impulsivity, inadequate decision-making, and impulsive–aggressive behaviour.

### 2.2. South African STATS on TBI and Return to Work

Moller, Lingah, and Phehlukwayo [1] stated that the prevalence of TBI within South Africa is 316 per 100,000 which is indicative of a high frequency rate in comparison to other countries.

According to Statistics South Africa [7], during the period from January 2014 to January 2016, a regional health facility in the Eastern Cape had a prevalence of 90 in 100,000 for TBI. Seventy-three percent of those persons with TBI were of working age. Data examined from a tertiary health facility in KwaZulu-Natal during the period from January 2009 to December 2013 revealed that severe brain injuries were attributed to 24 in 100,000 [7]. However, at the national level within South Africa, the current incidence and severity of TBI are unknown. Bearing in mind that the majority of individuals with TBI are the appropriate age for employment and fulfil the employee role, a leave of absence from work or the failure to resume work might have a significant effect on the economy.

### 2.3. Infection Risks for Patients and Rehabilitation Professionals During the COVID-19 Pandemic

According to Kleinitz et al. [8], as with other health services, in-person rehabilitation constitutes an infection risk that needs to be balanced alongside the consequences to patient outcomes and health facilities related to terminating or decreasing rehabilitation. Recommendations on how rehabilitation services are implemented during the pandemic would have to reduce exposure for clients and rehabilitation practitioners, particularly those who have a high probability of severe COVID-19 disease as a result of their age or comorbidities. The magnitude of risk will vary based on the accessibility of personal protective equipment (PPE) and additional infection prevention methods, which may differ throughout service delivery sites. Recommendations should also consider the likelihood of unconventional methods of rehabilitation, namely, telehealth, that could be applicable to certain types of rehabilitation, specifically those based on training and guidance. The feasibility of telehealth depends on local telecommunications infrastructure and the affordability of internet data for different socioeconomic groups, among other local factors [8].

### 2.4. The Impact of Rehabilitation Cessation or Reduction on Patient Outcomes During the COVID-19 Pandemic

As stated by Kleinetz et al. [8], rehabilitation services for specific patients who are not infected with COVID-19 ought to be recognised as essential services and should continue throughout the pandemic. Evidence suggests that reduction or cessation of rehabilitation services for patients with TBI, cerebrovascular accidents, burns, major surgery, spinal cord injury, myocardial infarction, and fractures could significantly influence the functional outcomes and health of these patients together with an increase in mortality.

Kleinitz et al. [8] suggest that extra effort should be made to ensure that rehabilitation services continue to operate throughout the pandemic as they contribute to the safe and prompt discharge from healthcare facilities for all patients, whether or not they have contracted COVID-19. When access to rehabilitation is suspended, it could result in prolonged hospital admissions, and inadequately planned or poorly organised rehabilitation could result in avoidable impediments and readmissions [8]. However, within the South African context and particularly at tertiary hospitals in Cape Town, South Africa, partial de-escalation of outpatient services was started on 20 March 2020, which meant that only certain outpatient clinics and services were functional, and patients were informed telephonically of a new appointment date [9].

### 2.5. Employment for a Person With a TBI

Statistics from an occupational therapy work assessment unit at a tertiary healthcare facility indicated that 97% of patients who suffered a mild or moderate brain injury were deemed unfit to return to employment within the labour force in South Africa [2].

According to Conklin, Flaumer, and Venables [10], vocational rehabilitation is considered a predictor for the resumption of work and maintenance of employment. The return-to-work process is significantly influenced by vocational rehabilitation [11]. Accessing rehabilitation services could facilitate the enhancement of residual symptoms and provide unconventional approaches to participating in the worker role [1].

Increased quality of life is commonly associated with resuming employment after TBI, and it is often considered an indication of favourable rehabilitation outcomes [12].

Liebson et al. [12] stated that return to employment is commonly deemed as the culmination of rehabilitation endeavours for rehabilitation service providers and individuals with TBI and is associated with several financial and psychological advantages. Employment following TBI is associated with enhanced quality of life, financial independence, and social integration. On the contrary, unemployment following TBI, coupled with low levels of productivity, is commonly related to poor quality of life, increased mental health issues, low self-confidence, and loss of financial independence and personal meaning.

## 3. Objective

The aim of the study is to explore the experiences and perceptions of TBI survivors about accessing rehabilitation during the COVID-19 pandemic and how this has affected their worker roles. In particular, the aims were (1) to explore the barriers TBI survivors experience when accessing rehabilitation and vocational rehabilitation during the COVID-19 pandemic and (2) to explore the facilitatory factors that aided TBI survivors when accessing rehabilitation and vocational rehabilitation services during the COVID-19 pandemic.

### 3.1. Research Team and Reflexivity

Interviews were conducted by the researcher (TP [Tarryn Petersen]), who is female and has a Bachelor of Science in Occupational Therapy (TP). The second author (MS [Mogammad Soeker]) is a male who has a PhD in occupational therapy. At the time that the study was conducted, Author 1 (TP) was a clinician working in an occupational therapy department at a tertiary hospital and Author 2 (MS) was the head of the occupational therapy department at a tertiary institution. Both researchers have experience in qualitative research, especially in conducting qualitative interviews, analysing findings, and disseminating the information. The researchers had no relationship with the research participants before the study commenced. The research participants did not have any engagement with the researchers; therefore, the process remained fair. The researchers provided information about the study and their motivation for conducting it in the information sheet of the study. The above was also verbally described to the research participants.

## 4. Design and Methods

For the purpose of this study, a descriptive, explorative qualitative research design was used. Qualitative research is defined as a research approach that occurs in a normal environment that allows the researcher to establish a level of detail from an in-depth participation in the actual experience [13]. In this study, qualitative research enabled the researcher to better understand the experiences and perceptions of clients who sustained a traumatic brain injury about accessing rehabilitation during the COVID-19 pandemic and how this affected their worker roles. The researchers of the current study also used a descriptive approach, in that it allowed the researchers to develop a detailed description of the contextual experiences of the participants about a study phenomenon [14]. In the current study, the use of descriptive research allowed the researcher to collect accurate, rich, and meaningful data from the participants, asking them to describe their experiences and perceptions about accessing rehabilitation throughout the COVID-19 pandemic and how this affected their rehabilitation and worker roles. The COREQ (consolidated criteria for reporting qualitative research) checklist was used as a reporting guideline.

## 5. Population and Sampling

Ten participants and two key informants were purposively sampled from the statistical records of the department of occupational therapy at a tertiary hospital within the Western Cape of South Africa. According to Cresswell and Plano Clark [15], purposive sampling requires finding and choosing individuals or groups of people who are particularly well-informed or knowledgeable with an experience of interest. The participants were carefully chosen from the statistical records of participants attending the occupational therapy department at a tertiary healthcare facility in the Western Cape. Furthermore, a set of inclusion and exclusion criteria was used to select participants from statistical records. The researchers used the following inclusion and exclusion criteria when selecting the participants. The inclusion criteria are as follows: (1) the participants had to have a confirmed diagnosis of a TBI in accordance with the Glasgow Coma Scale. (2) They must have sustained the TBI prior to or during the COVID-19 pandemic. (3) The participants would be either male or female, between the ages of 18 and 65 years, and be able to converse well in English, Afrikaans, or isiXhosa. (4) Participants must have either received or are in the process of accessing rehabilitation and vocational rehabilitation services during the COVID-19 pandemic. The inclusion criteria for key informants are as follows: (1) occupational therapists had to have at least 1 year of experience in TBI rehabilitation and vocational rehabilitation and they had to have provided rehabilitation services to clients during the COVID-19 pandemic.

The exclusion criteria are as follows: (1) TBI survivors who could not communicate in English, Afrikaans, or isiXhosa; participants who were displaying active acute psychiatric symptoms; and participants who were younger than the age of 18 years. The criteria of exclusion for key informants were (1) occupational therapists who had less than 1 year's experience in TBI rehabilitation and vocational rehabilitation and who did not provide rehabilitation services to clients during the COVID-19 pandemic.

## 6. Data Collection

Potential participants were identified with the assistance of occupational therapists at Groote Schuur Hospital. The potential participants were then contacted telephonically to determine whether or not they met the inclusion criteria of the study. The researcher arranged appointments with appropriate participants in order to discuss participation in the study. Upon meeting each participant, the aim and intentions of the study were explained verbally as well as in writing. Informed consent from each of the participants was then obtained, and thereafter, dates for the interviews were arranged. Ten participants and two key informants were part of the study (see Tables 1 and 2 for participants' demographic characteristics). Ten participants were sampled between the ages of 21 and 55. Only two of the participants were females, and the remaining eight participants were males. The two key informants were two coloured females between the ages of 35 and 45. None of the participants dropped out of the study. Data was collected from July to November 2021, and the researcher made use of an audio recorder to record the data. A pilot study was done prior to the data collection in order to determine if the semistructured interview questions were appropriate. For the purpose of the pilot study, the researcher discussed the semistructured interview guide with the supervisor, and thereafter, the semistructured interview guide was trialled with one participant. After the first interview was completed, the researcher discussed the feedback from the interview with the supervisor, and it was deemed that no changes needed to be made to the semistructured interview questions; therefore, the researcher proceeded with the rest of the interviews with the participants. A total of 12 in-person interviews were conducted. Ten interviews were conducted with the participants, and two interviews were conducted with the key informants. In addition to the above, a member-checking interview was conducted with each participant, resulting in a total of two interviews with each participant. This allowed participants to comment and/or provide corrections to their transcripts. The researcher asked each participant and key informant a variety of questions, with the aid of an interview guide as well as probes that were directly related to the objectives of the study. However, interviews were conducted with all participants and key informants until saturation of the data was achieved. These interviews were all completed by the researcher at a place accessible to the participant and the researcher (i.e., at the tertiary hospital). The interviews required that the researcher either conduct face-to-face interviews or telephonic interviews. However, all the participants and key informants opted for in-person interviews. To maintain confidentiality and abide by social distancing laws due to the COVID-19 pandemic, only the researcher and participant were present during the interviews. The duration of the interviews was approximately 30–45 min, and the researcher also made field notes during the interviews. No repeat interviews were done. All the interviews were conducted face-to-face. Strict COVID-19 infection prevention protocols were followed to minimize the risk of infection for the participants, key informants, and the researcher.

## 7. Data Analysis and Trustworthiness

The researcher utilized a data analysis method by Braun and Clarke [16] which has six phases. During the first phase, the researcher read the transcriptions numerous times to become familiar with the experiences and perceptions of the participants. In the second phase, the researcher (TP) analysed the participant's quotes line-by-line and was then able to identify codes that were similar in the entire data set. The coded data was further analysed and arranged into categories that had common meanings relating to the research aim. During the third phase, the researcher extracted relevant data and grouped the codes into associated categories and themes. Software was not used to manage the data during this study. In the fourth phase, the researcher reviewed the themes. Similar descriptions of experiences as well as actual quotes that arose during line-by-line coding of the data were extracted and clustered collectively to develop themes. In the fifth phase, the researcher refined each theme in relation to the overall meaning of the analysis and generated a clear definition and name for each theme. During the last phase, the researcher explored and analysed the various themes in relation to one another, the research question, and applicable literature. The researcher used strategies such as credibility, transferability, dependability, and confirmability to ensure the trustworthiness of the data. Credibility was ensured in this study by depicting the fundamental nature of the participants' natural manner of speaking, incorporating sighs, repetitions, and pauses in sentences. Transferability was attained by presenting thorough interpretations of the research techniques, participants and their backgrounds, and comprehensive accounts of the lived experiences of the participants. Dependability was accomplished by providing a dense account of the research techniques. The researcher utilized a data audit trail, which allowed other researchers to assess the findings of the study to determine whether they would reach similar interpretations of the findings, and in doing this, dependability was ensured. Triangulation and peer examination were also used to ensure dependability. Confirmability was achieved by using techniques such as peer evaluations, audit trails, and member checking, in addition to reflexivity, to impart a better understanding of her influence on the research information. The researcher also made use of a reflexive journal to document any personal experiences, interactions, and thoughts which allowed the researcher to reflect on her own preconceived ideas or personal biases which could have swayed the research findings [16].

### 7.1. Ethics Statement

The participants of this study were informed about the requirements of the study verbally, and written information was provided. The participants voluntarily consented verbally and in writing before they participated in the study. The study was approved by the Institutional Review Board of the University of the Western Cape (ethics number: BM21/4/6).

## 8. Results

The data analysis revealed three themes related to the barriers and facilitators to accessing rehabilitation during the COVID-19 pandemic. The following themes are discussed:

• Theme 1: The barriers to accessing rehabilitation during the COVID-19 pandemic.
• Theme 2: A lack of rehabilitation impacted on the individual with TBI occupational performance.
• Theme 3: The factors that facilitated access to rehabilitation during the COVID-19 pandemic.

### 8.1. Theme 1: The Barriers to Accessing Rehabilitation During the COVID-19 Pandemic

Theme 1 represents the participant's barriers to accessing rehabilitation programmes throughout the COVID-19 pandemic. The essence of the theme emphasises the impact that the suspension of rehabilitation services during the COVID-19 pandemic had on the participants. The categories were then further divided into subcategories.

#### 8.1.1. Category 1: Suspension of Rehabilitation Services due to the COVID-19 Pandemic

This category conveys the participant's descriptions of their perceptions of the suspension of OT rehabilitation services throughout the COVID-19 pandemic and how this has impacted them.

I think it's the fact that this is protocol, so we weren't supposed to bring patients in…the thing with cognitive therapy is that if you don't jump on it fast, or immediately then the gains that you get even if its small gains it doesn't happen easily after a period. So, with the limitations that the institution put in place in terms of us not being able to book patients… I think was a big factor. (K2)

#### 8.1.2. Category 2: Fear of Contracting COVID-19 When Accessing Rehabilitation and Engaging in Worker Role

In this category, participants expressed their fears about resuming employment during the COVID-19 pandemic. Even though wearing a mask within the workplace is mandatory, one participant explained that his colleagues are not very diligent with wearing their masks in the workplace.

I am scared because there at my work there aren't people who want to wear masks. (P7)

Participants voiced their concerns about attending hospital appointments during the pandemic as they feared that they would contract COVID-19 at the hospital.

I had to hire someone to take me here(hospital)… because I was scared. As I still am scared of the pandemic…Because it is real…I'm learning to distance myself from people. Which is one of the laws we have now. So ja, it's been difficult. (P1)

#### 8.1.3. Category 3: Transport Issues That Affected Access to Rehabilitation

The participants described that certain forms of public transport were suspended during the pandemic, which resulted in them having to make alternative transport arrangements and paying excessive amounts of money in order to travel to the hospital.

Ja (yes), because there are no taxis now nothing to come and there are also no busses. Now I must hire a motorbike…and the money is a lot to come here coz I must pay R200 coz there is no taxis. (P3)

### 8.2. Theme 2: Lack of Rehabilitation Negatively Influenced the Individual With TBI Occupational Performance

Theme 2 describes how the lack of OT rehabilitation during the COVID-19 pandemic impacted the participant's quality of life. It also describes the sense of loss of engagement in occupational roles as a result of the residual deficits that the participants are experiencing.

#### 8.2.1. Category 1: Residual Physical, Cognitive, and Psychological Deficits Affecting the Individual With TBIs Quality of Life

Yes, my arms and hands are still very weak now…I can't fix my house anymore…like if there is something broken in the house, I can't fix it anymore. (P3)

A participant emphasised how a lack of cognitive rehabilitation post-TBI influences their ability to participate in meaningful occupations.

If we are talking about cognition specifically, I think it's more the attention and the memory and it just seems like their processing is slow. So, in order for them to think on their feet, for them to remember to do the task that they are supposed to do at work…it's just a problem. And then also executive function in terms of thinking on their feet. Uhm flexible thinking…they tend to lack that. (K2)

#### 8.2.2. Category 2: A Sense of Loss of Engagement in Occupational Roles

Participants describe their experiences of not being able to perform meaningful ADLs. Not being able to complete household chores due to residual physical symptoms resulted in participants experiencing a sense of loss in occupational engagement and questioning their self-worth.

I try to bring my part also in the home…like washing up dishes and stuff but every day I break something. Because of the left hand that is weak. I never used to let my wife iron my clothes. That I used to do myself but now she has to do that for me. (P5)

Man, I won't be able to work again. I won't be able to drive again. It's very difficult because I can have a fit or a seizure or a stroke while I am driving…and I am a truck driver. (P6)

I haven't been able to go to work since. So that's also been a big thing. And I think that has been also because my boss sees me as not being fit or as fit as I used to be. (P9)

### 8.3. Theme 3: Factors That Facilitated Access to Rehabilitation During the COVID-19 Pandemic

This theme aims to describe the factors that facilitated access to OT rehabilitation services during the COVID-19 pandemic.

#### 8.3.1. Category 1: Finding Innovative Ways of Enabling Individuals With TBI to Engage in Rehabilitation During COVID-19 Pandemic

The use of telemedicine/telehealth became a very popular form of treatment throughout the COVID-19 pandemic as it allowed health professionals to provide intervention while abiding by the lockdown regulations (i.e., social distancing). Participants describe how they used various forms of telemedicine/telehealth in order to provide OT intervention to TBI survivors as well as provide education and support to families of TBI survivors.

I've been using a lot of the ACLS (Allen Cognitive Level Screen Test) with the patients and then I phone the family. Simply to give the family a guide of what they gonna require. Because I think that's been a big thing like they don't know what to expect. So that's what I've been doing. And I think like during discharge within the COVID period, it's just escalated the amount of times I've done that. (K2)

Participants describe how the use of social media platforms assisted with sending OT intervention videos as well as home programmes to TBI survivors during the COVID-19 pandemic.

So even now just with the level 4 restrictions and the hospital having to cut visiting times. So, what we did in the department was… the videos…Sending through videos to family members in terms of transfers and how to do passive movements and just like sort of pressure care and things like that. So that has been a big thing in the department. (K2)

Home programmes have been developed so that patients…when they are not able to access the care then they are able to access… the therapeutic intervention that they would have required. Obviously not overseen by a therapist but at least it's better than nothing. (K1)

#### 8.3.2. Category 2: Occupational Therapists' Ability to Triage Rehabilitation Services to Maximise Rehabilitation Benefits to Individuals With TBI During the COVID-19 Pandemic

In order to limit exposure for clients deemed at high risk of contracting COVID-19, OTs needed to triage clients before deciding to do a face-to-face intervention. During the COVID-19 pandemic, OTs in the study setting also triaged clients who were employed in the open labour market to ensure that they received rehabilitation in order to maintain their employment. One participant said:

The activities that she is doing with me I believe they are preparing me to be in a position where… when I get back to my kiddies, then I will be ready to teach them again. (P10)

I think it wasn't too long coz it was only a few weeks…. She is showing me how to do work and do my job again. She does exercises with my hand and my leg so that when I go back to work, I will be able to pick up the boxes again. …and she say I must come back for more sessions. (P3)

## 9. Discussion

### 9.1. Barriers Related to Accessing Rehabilitation During the COVID-19 Pandemic

#### 9.1.1. Suspension of Occupational Therapy Rehabilitation Services

According to Bettger et al. [17], rehabilitation services that enhance cognitive and physical performance to decrease disability are an essential constituent of high-level care. Category 1 of Theme 1 expressed the participants' experiences and perceptions relating to the suspension of rehabilitation services during the COVID-19 pandemic. It was found that the suspension of rehabilitation during the COVID-19 pandemic negatively influenced the participant's ability to access rehabilitation which had a direct negative influence on their ability to resume their worker roles. Although these choices were justified for the purpose of the protection of both the general public and healthcare practitioners, the consequences were a rise in disability and illness as a result of a deficiency of essential rehabilitation care for those who require ongoing care. Deledda et al. [18] state that healthcare institutions and care facilities have implemented different processes in an attempt to limit the spread of COVID-19, particularly among patients with comorbidities. These techniques consist of suspension of medical visits, confinement of patients, rehabilitation, and follow-ups that are considered inessential services [18]. Participants in this study expressed that the de-escalation of nonessential services negatively impacted them to the extent that they are now experiencing functional limitations which could have been addressed if they were allowed access to occupational therapy rehabilitation services during the COVID-19 pandemic.

#### 9.1.2. Fear of Contracting COVID-19

Deledda et al. [18] note that frequently, patients were contacted by healthcare facilities to suspend or cancel booked appointments, therapeutic interventions, and/or surgical procedures to limit the chances of becoming infected with COVID-19. On the other hand, patients have subsequently contacted healthcare facilities to cancel appointments [18]. This was evident in the current study as participants expressed their concerns about attending hospital appointments during the pandemic as they feared that they would contract COVID-19 at the hospital. The participant's unique reports and perceptions of contracting the virus while attending their medical appointments often resulted in the participants choosing not to attend their medical appointments. Undeniably, the workplace has some aspects that are likely to accelerate the increase in COVID-19 infections. Participants in the current study indicated that even though wearing a mask within the workplace was mandatory, their colleagues were not very diligent about wearing their masks in the workplace, and this in turn contributed to the fears of the participants about resuming employment throughout the COVID-19 pandemic.

#### 9.1.3. Lack of Rehabilitation Negatively Influences Quality of Life

Polinder et al. [19] state that health-related quality of life (HRQoL) describes a person's view on how a medical condition and its treatment influence physical, psychological, and social factors of their life. In this study, it was found that the suspension of rehabilitation services during the COVID-19 pandemic had resulted in the participants still experiencing residual physical, cognitive, and psychological deficits.

Numerous studies have found that achieving independence in ADLs and IADLs together with adequate social support and a fulfilling job promotes increased quality of life following TBI [20]. In accordance with the findings of the current study, it was found that participants not being able to access rehabilitation during the pandemic, coupled with them not being able to complete self-care tasks and household chores due to residual physical symptoms, resulted in participants experiencing a sense of loss in occupational engagement and the questioning of their self-worth.

#### 9.1.4. Transport Issues During the COVID-19 Pandemic Creating Barriers to Access Occupational Therapy Rehabilitation

Cochran [21] found that barriers related to access to transport to medical facilities often cause missed appointments or postponement of care, which excessively affects persons with disabilities. Furthermore, it was found that individuals with disabilities experience issues with transportation to healthcare more frequently than persons without disabilities, and it is often regarded as a social element of health [21]. Deledda et al. [18] note that during the SARS-CoV-2 outbreak, patients' reasons for cancelled appointments included being unable to get time off work, secure childcare, and/or find a safe mode of transport [21]. In the current study, participants expressed how the public transport system had also been impacted by the COVID-19 pandemic to the extent that certain forms of public transport were suspended during the pandemic, which resulted in participants having to make alternative transport arrangements and paying excessive amounts of money in order to travel to the hospital for their rehabilitation appointments. In an effort to curb the risk of transmission of COVID-19, governments have implemented restrictions on travel and adapted a work-from-home policy for nonessential employees, subsequently affecting transport usage. This has impacted all modes of transportation including railways, airlines, subway systems, and buses which have all experienced an unparalleled decrease in patrons. Gkiotsalitis and Cats [22] state that due to the increase in the number of COVID-19 infections, many countries employed social distancing protocols in places of employment, schools, shopping centres, and public transport. These measures have had a significant influence on public transport services and service delivery [22]. This is evident in the current study, as COVID-19 restrictions with regard to social distancing mean that public transport operators were only allowed to load a certain number of patrons, resulting in lengthy waiting times for public transport in order for participants to travel to their OT rehabilitation appointments.

#### 9.1.5. Lack of Rehabilitation Impacts on Resumption of Worker Role

Materne, Lundqvist, and Strandberg [23] assert that the resumption of employment influences an individual's self-esteem, quality of life, and well-being and results in the individual experiencing a state of normality in civilisation and a measure of success. Moller, Lingah, and Phehlukwayo [1] found that resumption of employment was better facilitated when some form of vocational rehabilitation was obtained. Participants conveyed their experience of loss of employment post-TBI as they were unable to return to work due to the de-escalation of rehabilitation services throughout the COVID-19 pandemic. However, due to participants not being able to attend outpatient OT rehabilitation, they were unable to resume their worker roles, which ultimately resulted in the loss of employment.

### 9.2. Facilitators Related to Accessing Rehabilitation During the COVID-19 Pandemic

#### 9.2.1. Telemedicine/Telehealth Enables the Continuation of Rehabilitation Services

According to Becevic et al. [24], telehealth offers the possibility of decreasing the number of trips to hospitals and doctors' consultation rooms. The decline in these visits assisted with curbing the transmission of COVID-19, specifically decreasing the rate of infections between COVID-19 positive patients and healthcare workers as well as safeguarding patients with comorbidities who are at risk of severe disease [25]. In the current study, it became evident that the use of telemedicine/telehealth became a very popular form of treatment throughout the COVID-19 pandemic as it allowed health professionals to provide intervention while abiding by the lockdown regulations (i.e., social distancing). The use of social media platforms assisted with sending OT intervention videos as well as home programmes to TBI survivors during the COVID-19 pandemic.

#### 9.2.2. The Use of Home Programmes Allowed for the Continuation of OT Rehabilitation

Makaram et al. [26] propose that home exercise programmes (HEPs) may be an excellent preferred alternative to formal or outpatient rehabilitation therapy sessions for clients during periods of COVID-19 lockdown or self-isolation, as they are cost-effective without compromising quality. This was apparent in the current study, where participants received OT home programmes as they were unable to access OT rehabilitation during the COVID-19 pandemic. After sustaining a TBI, many survivors experience significant cognitive limitations. Thus, cognitive intervention plays a vital role in TBI rehabilitation [27]. Participants expressed how a home programme, specifically designed for cognitive intervention for TBI survivors, assisted participants with being able to continue rehabilitation during the COVID-19 pandemic.

#### 9.2.3. Triaging Rehabilitation Services to Maximise Rehabilitation Benefits to Individuals With TBI During the COVID-19 Pandemic

Harding, Taylor, and Shaw-Stuart [28] found that allied health clinicians, including occupational therapists, often needed to make decisions regarding the comparative urgency of referrals, prioritise patients for treatment, and, in some instances, determine whether patients need services at all. The words “triage” and “prioritisation” illustrate procedures or tools that assist clinicians in the provision of their services. The objective of these procedures may be to determine which patients need to be prioritised, to designate patients according to how services could be offered (by OTs or assistants, for example), or to decide who needs treatment at all [28]. In Theme 3, Category 2, it was found that fulfilling the worker role is deemed a meaningful occupation as it allows TBI survivors to continue to provide for themselves and their families. The findings of the current study found that occupational therapists in the study setting also triaged clients who were employed in the open labour market to ensure that they received optimal rehabilitation in order to maintain their employment.

## 10. Limitations of the Study

The current study has several limitations. Some participants struggled to articulate themselves during data collection, which could be attributed to the residual effects of their TBI. Secondly, only two of the study participants were female, whereas the remaining eight were male. The two female participants reported a range of unique difficulties (e.g., psychological symptoms and impact on quality of life) that they, as women, experienced after their TBI, whereas most of their male counterparts did not mention this. Thirdly, due to time limitations and COVID-19 infection prevention protocols, some of the interviews were conducted after the participants had completed their medical appointments. This may have had an impact on the quality of the data collected during the interviews as participants could have been experiencing fatigue and thus provided superficial or less meaningful descriptions of their experiences and perceptions. Fourthly, some of the member-checking interviews were conducted virtually and not face-to-face. Therefore, poor internet connectivity may have caused interrupted communication. Lastly, all the participants came from one hospital, therefore restricting the ability of the study findings to be generalised to other settings.

## 11. Conclusion

The study found that there were barriers and facilitators to accessing occupational therapy rehabilitation during the COVID-19 pandemic for TBI survivors. The barriers comprised mostly of external factors within the environment, which impacted TBI survivors' ability to access occupational therapy rehabilitation during the COVID-19 pandemic. The study also highlighted how the scarcity of rehabilitation influenced TBI survivors' quality of life and led to the loss of their worker roles. The facilitators comprised innovative and alternative methods used by occupational therapists throughout the COVID-19 pandemic to ensure that TBI survivors had access to occupational therapy rehabilitation while still adhering to national lockdown restrictions and COVID-19 infection prevention strategies.

The current study found that, as much as the COVID-19 pandemic created barriers to accessing occupational therapy rehabilitation, it also encouraged occupational therapists to develop innovative ways of providing rehabilitation to TBI survivors. The study also provided insight into the effectiveness and value of occupational therapy home programmes for TBI survivors. Furthermore, this study revealed that telehealth/telemedicine can be used as an effective method of outpatient occupational therapy rehabilitation if a TBI survivor is unable to attend an in-person rehabilitation appointment. Overall, this study highlighted the value of occupational therapy rehabilitation services for TBI survivors as they directly influence participation in meaningful occupations as well as the resumption of the worker role.

## Figures and Tables

**Table 1:** Participants' demographic characteristics.

**Participants**	**Gender**	**Age**	**Date of injury**	**Work experience/job title**	**Rehab received during data collection period**
P1	Male	23	November 2020	Trolley porter	Yes
P2	Female	55	June 2021	Seamstress	Awaiting rehab appointment
P3	Male	33	July 2021	General worker—dispatch	Yes
P4	Male	28	October 2019	Cleaner	Awaiting rehab appointment
P5	Male	46	July 2021	General worker—dispatch	Awaiting rehab appointment
P6	Male	37	December 2020	Long distance truck driver	Yes
P7	Male	21	July 2021	General worker—installing aluminium windows	Yes
P8	Male	31	June 2021	Teacher	Awaiting rehab appointment
P9	Female	22	June 2021	Security officer	Yes
P10	Male	37	July 2021	Teacher	Yes

**Table 2:** Key informants' demographic characteristics.

**Key informant**	**Gender**	**Age group**	**Designation**	**Current work**	**Experience working with TBI survivors**
KI-1	Female	35–40	Occupational therapist	Tertiary healthcare facility	10 years
KI-2	Female	40–45	Occupational therapist	Tertiary healthcare facility	12 years

## Data Availability

The data that support the findings of this study are available from the corresponding author upon reasonable request.
